# Variability of functional traits and their syndromes in a freshwater fish species (*Phoxinus phoxinus*): The role of adaptive and nonadaptive processes

**DOI:** 10.1002/ece3.4961

**Published:** 2019-02-14

**Authors:** Allan Raffard, Julien Cucherousset, Jérôme G. Prunier, Géraldine Loot, Frédéric Santoul, Simon Blanchet

**Affiliations:** ^1^ CNRS, UMR‐5321, Station d’Écologie Théorique et Expérimentale du CNRS à Moulis Université Toulouse III Paul Sabatier Moulis France; ^2^ EcoLab, Université de Toulouse CNRS, INPT, UPS Toulouse France; ^3^ CNRS, UMR‐5174 EDB (Laboratoire Evolution & Diversité Biologique) Université Toulouse III Paul Sabatier Toulouse France

**Keywords:** adaptation, freshwater fish, genetic drift, intraspecific variation, rivers, syndrome, trait covariation

## Abstract

Functional traits can covary to form “functional syndromes.” Describing and understanding functional syndromes is an important prerequisite for predicting the effects of organisms on ecosystem functioning. At the intraspecific level, functional syndromes have recently been described, but very little is known about their variability among populations and—if they vary—what the ecological and evolutionary drivers of this variation are. Here, we quantified and compared the variability in four functional traits (body mass, metabolic rate, excretion rate, and boldness), their covariations and the subsequent syndromes among thirteen populations of a common freshwater fish (the European minnow, *Phoxinus phoxinus*). We then tested whether functional traits and their covariations, as well as the subsequent syndromes, were underpinned by the phylogenetic relatedness among populations (historical effects) or the local environment (i.e., temperature and predation pressure), and whether adaptive (selection or plasticity) or nonadaptive (genetic drift) processes sustained among‐population variability. We found substantial among‐population variability in functional traits and trait covariations, and in the emerging syndromes. We further found that adaptive mechanisms (plasticity and/or selection) related to water temperature and predation pressure modulated the covariation between body mass and metabolic rate. Other trait covariations were more likely driven by genetic drift, suggesting that nonadaptive processes can also lead to substantial differences in trait covariations among populations. Overall, we concluded that functional syndromes are population‐specific, and that both adaptive and nonadaptive processes are shaping functional traits. Given the pivotal role of functional traits, differences in functional syndromes within species provide interesting perspectives regarding the role of intraspecific diversity for ecosystem functioning.

## 1 INTRODUCTION

Phenotypic variability measured within species has historically been the core of evolutionary studies, as it constitutes the visible outcome of evolutionary processes (Darwin, 1859; Roff, 1992; Stearns, 1992). It is now increasingly acknowledged that intraspecific phenotypic variability can strongly affect community structure and ecosystem functioning (Des Roches et al., 2018; Raffard, Santoul, Cucherousset, & Blanchet, 2018). In particular, functional traits, such as excretion rate, are extremely important for understanding and predicting how organisms affect their own biotic and abiotic environment (Díaz et al., 2013; Violle et al., 2007). Functional traits display variability both within and among populations (Helsen et al., 2017; Villéger, Brosse, Mouchet, Mouillot, & Vanni, 2017). For instance, the nutrient excretion rate (a trait potentially affecting nutrient availability in ecosystems, Vanni, 2002) can vary substantially among and within populations (Evangelista, Lecerf, Britton, & Cucherousset, 2017; Villéger, Grenouillet, Suc, & Brosse, 2012). Since functional traits determine the way organisms modulate the environment, it is important to investigate the spatial distribution of these traits (Funk et al., 2016; Villéger et al., 2017).

Functional traits are highly variable across landscapes. For instance, the metabolic rate of ectotherms is, on average, higher in warm than in cold environments (Brown, Gillooly, Allen, Savage, & West, 2004; Hildrew, Raffaelli, & Edmonds‐Browns, 2007). Moreover, trait covariations are also expected to be heterogeneous across landscapes (Reale et al., 2010). The covariations among multiple traits have been referred to as *syndromes*(Dingemanse et al., 2007). Syndromes have primarily been investigated for life‐history, behavioral, and physiological traits (Roff, 1992; Sih, Bell, & Johnson, 2004), and have greatly contributed to our understanding of life‐history strategies in wild populations (Reale et al., 2010). In the meantime, community ecologists have investigated how covariations in functional traits, measured at the community level, can affect ecosystem functioning (Díaz et al., 2016; Lavorel & Garnier, 2002). More recently, it has been demonstrated that functional trait covariations also occur within species, forming a so‐called *functional syndrome* (Raffard et al., 2017). Functional syndromes have been shown to exist in several species (e.g., Defossez, Pellissier, & Rasmann, 2018; Raffard et al., 2017), but the variability of these syndromes across populations and environmental conditions remains unexplored.

Functional syndromes are also expected to vary among populations within a single species (Peiman & Robinson, 2017). Indeed, it has been suggested that environmental conditions can modulate trait covariations and the associated syndromes (Killen, Marras, Metcalfe, McKenzie, & Domenici, 2013). Notably, experimental studies have demonstrated that some environmental conditions can induce biological constraints (e.g., energetic requirement) that modulate trait covariations (Finstad, Forseth, Ugedal, & NæSje, 2007; Killen, Marras, & McKenzie, 2011). For instance, food availability has been demonstrated to produce a covariation between metabolic rate and risk‐taking behavior in European sea bass (*Dicentrarchus labrax*), probably because individuals with high metabolic rate have high energetic demands and should be more active to acquire resources to sustain this demand (Killen et al., 2011). Variation in syndromes has also been reported among wild populations living in heterogeneous environments (Dingemanse et al., 2007; Peiman & Robinson, 2012; Pruitt et al., 2010; Závorka et al., 2017). Beyond the direct effect of environmental characteristics (e.g., temperature, predation) on syndromes, the evolutionary history of populations—such as the past demographic and colonization history that often generates bottlenecks and founder effects—may also play an underestimated role in shaping syndromes (Armbruster & Schwaegerle, 1996; Peiman & Robinson, 2017). For instance, populations can exhibit different syndromes because they may have been colonized by two independent lineages having evolved divergent syndromes in their past respective refuge (“the ghost of colonization past”). This past evolutionary legacy is likely to be identified at the level of the genetic lineages; two closely related populations being more likely to display similar syndromes than two distantly related populations. This possible evolutionary legacy of syndromes has—up to our knowledge—rarely been considered.

In this study, we investigated the variability of functional traits and the syndromes they form in wild populations inhabiting heterogeneous environments. Using a common freshwater fish (the European minnow, *Phoxinus phoxinus*) as a model species, we aimed at testing (a) whether functional traits and their covariations vary between populations, and (b) whether this variability is explained by environmental factors and/or the evolutionary history of populations. Focusing on four functional traits (i.e., excretion rate, metabolism, body mass, and boldness), we first expected that both mean values and covariations of traits differ between populations because of their contrasting environments and evolutionary histories. Second, we focused on two environmental characteristics (temperature and predation intensity) that affect functional traits (e.g., metabolism, Gillooly, 2001), and that are hence likely to also modulate their covariations. Temperature is indeed a key abiotic factor for ectotherms as it can affect their metabolic rate, behavior, and body mass (Biro, Beckmann, & Stamps, 2010; Brown et al., 2004; Gillooly, 2001). Additionally, predation risk can affect the physiology and behavior of individuals by inducing strong stresses (Bell & Sih, 2007; Hawlena & Schmitz, 2010). We concomitantly tested the contribution of the past evolutionary history of populations to explain variation in covariations among functional traits using phylogenetic models. Specifically, we assessed the relationships between genetic similarity (inferred from microsatellite markers) and syndrome similarity among populations. An influence of the environment on traits would suggest potential adaptation (or plasticity of these syndromes), and we hence finally used a quantitative genetic approach (*P*_ST_/*F*_ST_, Leinonen, McCairns, O'Hara, & Merilä, 2013) to infer the evolutionary processes (genetic drift vs. selection/plasticity) underlying differences in trait variation and covariation among populations.

## 2 MATERIALS AND METHODS

### 2.1 Model species

The European minnow (*P. phoxinus*) is an abundant species in Western Europe in cold lakes (e.g., mountains lakes) and rivers (e.g., from small rivers at intermediate altitude to mountain streams) with summer water temperature generally lower than 22–24°C (Keith, Persat, Feunteun, & Allardi, 2011). It is a small‐bodied fish species (<12 cm long, 5–8 cm long as an adult in general) living approximately 3 to 5 years, and which displays a generalist diet composed of small invertebrates, algae, or zooplankton (Collin & Fumagalli, 2011; Frost, 1943). The European minnow is considered as a genotypically and phenotypically variable species (Collin & Fumagalli, 2011, 2015; Fourtune et al., 2018).

### 2.2 Sampling sites and animal rearing

**Figure 1:**
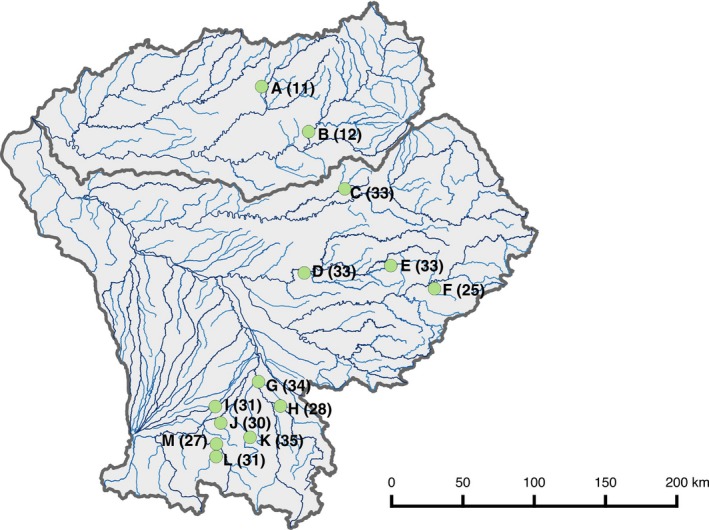
Distribution of the thirteen studied populations of European minnows (*Phoxinus phoxinus*). Names of populations were coded from A to M, and the number of individuals for each population is given as indication

We focused on riverine European minnow populations from the Dordogne–Garonne river basin in southwestern France (Figure 1). We selected thirteen sites (coded from A to M) in different rivers to reflect their potential colonization history (Fourtune, Paz‐Vinas, Loot, Prunier, & Blanchet, 2016; Paz‐Vinas et al., 2018). Sampled rivers were selected based on previous knowledge in terms of environmental and geographic characteristics of the area (Fourtune et al., 2016, 2018).

For each site, we focused and measured two environmental variables that have been shown to modulate functional traits in ectotherms (Bestion, Teyssier, Aubret, Clobert, & Cote, 2014; Biro et al., 2010; Gillooly, 2001), and hence potentially their covariations. We first recorded water temperature, which was measured as the mean temperature between July and September 2017, using automatic sensors (HOBO®, one measure every hour). Mean summer water temperature varied from 15.5°C (site E) to 21.5°C (site D) (Figure 1). In addition, we measured the local predation pressure, a key biotic factor that can affect organisms’ phenotype (Langerhans, 2007). Predation pressure was calculated for each site as the density of piscivorous fishes (namely northern pike, *Esox lucius*; brown trout, *Salmo trutta*; rainbow trout, *Oncorhynchus mykiss*; European perch, *Perca fluviatilis;*pikeperch*, Sander lucioperca*; and European eel, *Anguilla anguilla*). This metric was similar to that described in Edeline, Lacroix, Delire, Poulet, and Legendre (2013). This index of predation was calculated by dividing the number of sampled predator individuals by the surface covered during sampling; these data—for each site—were sourced from Fourtune et al. (2016) and from the French Agency for Biodiversity (Poulet, Beaulaton, & Dembski, 2011).

In summer 2016, we collected adult fish on these thirteen sites using electrofishing (Figure 1). On each river, we collected approximately one hundred adults along a ~200‐m‐long river stretch to ensure representativeness of the fish habitat. Then, we randomly sampled 30–40 individuals among the sampled adults to have a representative subsample of each population. Electrofishing was performed under the authorization of “Arrêté Préfectoraux” delivered by the “Direction Départementale des Territoires” of each administrative department (Haute‐Garonne, Ariège, Aveyron, Lot, Tarn and Corrèze). Laboratory rearing of fish was performed under authorizations of the “Direction Départementale de la Cohésion Sociale et de la Protection des Populations (service Santé Protection des Animaux et Environnement) de l'Ariège,” Arrêté Préfectoraux SA‐013‐PB‐092 and Certificat de Capacité 09‐273. Fish were brought to the laboratory and maintained in a thermoregulated room for two to four weeks before experiments. Fish from the different populations were held in independent 150‐L tanks in which water temperature was set to 17°C and photoperiod to a light:dark cycle of 12:12 (Golovanov, 2013). They were fed with frozen bloodworms three times a week. Prior to experiments, fish were anesthetized (benzocaine, 25 mg/L), weighed (to the nearest 0.01 g), and tagged with a Passive Integrated Transponder (PIT) tags (8 × 1.4 mm, FDX‐B “skinny” PIT tag, Oregon RFID, USA) inserted in the general cavity using a sterile scalpel. Fish recovered and acclimatized to the rearing room for 10 days before the quantification of three functional traits in addition to body mass (boldness, excretion rate, and metabolic rate). Metabolic rate was measured on day 1 (morning), while excretion rate and boldness were measured on day 2 in the morning and in the afternoon, respectively. Before quantifying metabolic rate, individuals were starved for two days to ensure the same starvation level among individuals.

### 2.3 Boldness

Boldness was assessed for each individual independently in circular containers (30 cm in diameter) filled with 5L of dechlorinated tap water at 17°C and 500 ml of water from a tank containing conspecifics. The containers were surrounded by curtains to standardize light conditions and to hide the experimenter. A shelter (pipe, 7 cm length × 3 cm diameter) was added in each container to allow the fish to hide. After having introduced each individual into the shelter and after 10 min of acclimatization to reduce stress level induced by handling, the shelter was opened and each individual was filmed for fifteen minutes. Video footage was subsequently analyzed with the software “BORIS” (Friard & Gamba, 2016). Boldness was quantified as the time spent outside of the shelter. The order and the containers in which individuals were assayed were randomly attributed. All behavioral assays were performed in the afternoon (from 12:00 p.m. to 16:00 p.m.) to minimize the potential effects of circadian rhythms.

### 2.4 Excretion rate

Excretion rate was quantified using nitrogen excreted by organisms as the dissolved form of ammonium ${\text{NH}}_{4}^{+}$. Quantifying excretion rate on starved individuals was done to avoid an effect of differential consumption, which is a strong factor affecting the rate of nitrogen excretion (Glaholt & Vanni, 2005). Changes in ${\text{NH}}_{4}^{+}$ concentration in water can affect ecosystem functioning through an increase in nutrient availability (Capps & Flecker, 2013) and primary production (Bassar et al., 2016; Schmitz, Hawlena, & Trussell, 2010). Following Villéger et al. (2012), individuals were placed in plastic bags containing 500 ml of spring bottled water for 1 hr at 17°C. Individuals were then removed and 100 ml of water was filtered through a glass microfiber filter (Whatman, GF/C, diameter = 25 mm), and samples were frozen at −20°C. Excretion rate (${\text{NH}}_{4}^{+}$ in μg L^−1^ hr^−1^) was determined with a high‐performance ionic chromatograph (Dionex DX‐120).

### 2.5 Metabolic rate

We measured the oxygen consumption rate as a proxy of the metabolic rate of individuals. Fish were individually placed in a custom made metabolic chamber filled with 500 ml of dechlorinated tap water and hermetically sealed. Chambers were set in a thermoregulated room at 17°C in the dark to lower the stress level. We measured the metabolic rate just after handling so that the same stress was imposed to all individuals. Measurements of oxygen concentration were taken after 10 min, allowing individuals to acclimate, and continuously every five seconds for 50 min using oxygen probes (OXROB10, Pyroscience). The metabolic rate was calculated as the absolute slope between oxygen quantity in the chamber and time, reflecting the hourly consumption of oxygen (mg/hr).

### 2.6 Genetic analyses

Thirty additional adults from each of the thirteen sites were sampled for genetic material. For each of these thirty individuals, we collected and preserved in 70% ethanol a small piece of pelvic fin and individuals were then released in their respective sampling site. Genomic DNA was extracted using a salt‐extraction protocol (Aljanabi, 1997). Eighteen autosomal microsatellite markers were considered in this study: Polymerase chain reactions (PCR) and genotyping were performed as detailed in Supporting Information Appendix S1, resulting in a final data set of 357 genotypes. We checked for multilocus deviation from Hardy–Weinberg equilibrium (HWE) and for gametic disequilibrium using GENEPOP 4.2.1 (Rousset, 2008) after sequential Bonferroni correction to account for multiple related tests (Rice, 1989). The presence of null alleles was then assessed at each locus by analyzing homozygote excess in five populations that did not follow HWE, using MICROCHECKER 2.2.3 (Van Oosterhout, Hutchinson, Wills, & Shipley, 2004). We discarded from further analyses any locus showing significant gametic disequilibrium and/or evidence of null alleles, resulting in the withdrawal of one locus (CtoG‐075), for a total number of seventeen loci.

We computed Nei's standard genetic distance (Nei, 1973) between each pair of populations using the *diveRsity* R‐package (function *diffCalc*; Keenan, McGinnity, Cross, Crozier, & Prodöhl, 2013). A hierarchical cluster analysis was then performed to uncover genetic relatedness among the thirteen populations using the functions *hclust* (R‐package *stats*) and *as.phylo* (R‐package *ape*; Paradis, Claude, & Strimmer, 2004) to convert the genetic dissimilarity matrix into an unrooted phylogenetic tree based on complete linkage method.

Finally, we estimated the overall level of genetic differentiation *F*_ST_ among the thirteen populations using the *hierfstat*R‐package (Goudet, 2005). The resulting global *F*_ST_ corresponds to the inter‐population variance component in allelic frequencies (Yang, 1998), and to the level of differentiation among populations due to genetic drift only (Leinonen et al., 2013). This value is directly comparable to the interpopulation variance component in quantitative traits (*P*_ST_, see below). A 95% confidence interval (CI) was computed for the observed global *F*_ST_ value using a classical cluster bootstrap procedure with 1,000 iterations (Field & Welsh, 2007): CI lower and upper bounds were computed as the 95% percentiles of a theoretical distribution of 1,000 *F*_ST_ values obtained from the random sampling of the thirteen populations with replacement.

### 2.7 Statistical analyses

#### 2.7.1 Trait variability among populations

For each of the four traits separately, we tested whether there was significant variability among the thirteen populations using an analysis of variance (ANOVA) with the population of origin as the explicative categorical variable. To meet the assumptions of Gaussian models (normality of the residuals and homoscedasticity), data were transformed: Body mass, metabolic rate, and excretion rate were log‐transformed and boldness was square‐root‐transformed.

#### 2.7.2 Heterogeneity in trait covariations among populations

We tested whether covariations among the four traits (i.e., syndromes) were different among the thirteen populations. We first synthetized and described, for each population, patterns of trait covariation using path analysis. Traits were scaled to the mean within each population (i.e., each population displays a mean of zero with a variance of one for each trait), and a general path analysis linking each trait to the others (saturated path analysis) was computed for each population independently using the *lavaan* R‐package (Rosseel, 2012). These resulted in thirteen path models (each path model corresponding to a population's syndrome) and thirteen associated covariance matrices. Then, we then tested whether these path models (and hence trait covariations) varied among populations using a test of heterogeneity on covariance matrices among groups (*metaSEM* R‐package, Cheung, 2015). Briefly, this analysis allows assessing the heterogeneity of covariance matrices with a combination of indices (Hooper, Coughlan, & Mullen, 2008): (a) root mean square error of approximation (RMSEA, expected to be higher than 0.06 if the matrices are heterogeneous), (b) standardized root mean square residual (SRMR, expected to be higher than 0.09 if the matrices are heterogeneous), and (c) comparative fit index (CFI, expected to be lower than 0.96 if the matrices are heterogeneous).

#### 2.7.3 Heterogeneity of pairwise covariations

We tested whether the six covariations considered separately differed among populations using a test of heterogeneity (Rosenberg, Adams, & Gurevitch, 1997). We estimated and extracted the covariations between each pair of traits (six pairs in total: mass–metabolism; mass–excretion; mass–boldness; metabolism–excretion; metabolism–boldness; and excretion–boldness) from the path models described above so as to control for all relationships among traits simultaneously. We applied meta‐analytic tools to analyze the heterogeneity in covariances. We applied the Z‐Fisher transformation to each covariance value (*Cov*) to obtain a standardized *Zr* using the following formula: $Zr=0.5\ln\frac{\left(1+Cov\right)}{\left(1-Cov\right)}$, and we calculated the corresponding standard error as: $se_{\mathrm{Zr}}=\frac{1}{\sqrt{n-3}}$ (Nakagawa & Cuthill, 2007) where *n* is the sample size of the considered population. We estimated the degree of variability of *Zr* for each pair of traits among populations with a test of heterogeneity (Higgins & Thompson, 2002; Viechtbauer, 2010). This index (*H*) indicates the percentage of heterogeneity and tests whether heterogeneity in a data set is higher than that expected by chance. The standard error of *Zr* was added as a pondering parameter to the heterogeneity test to give more weight to populations with more individuals.

#### 2.7.4 Effect of phylogeny

We tested whether phylogenetically related populations displayed similar traits and trait covariations using phylogenetic models (PGLS, Garland & Ives, 2000). These models allow incorporating the genetic relatedness among populations through a phylogenetic tree used to estimate a λ value corresponding to the degree of phylogenetic conservatism in the response variable. λ is expected to vary between 0 and 1, where 0 means no phylogenetic dependence in a trait among populations, and 1 means that the focal trait is phylogenetically conserved (Comte, Murienne, & Grenouillet, 2014; Harvey & Purvis, 1991). We calculated λ independently for each trait and each covariation (calculated from path analyses; see above) using only the intercept as fixed effect.

#### 2.7.5 Effect of environmental characteristics

We used phylogenetic models to assess the effects of temperature and predation on traits and covariations. We ran PGLS for each trait and covariation (*Zr*) independently, with temperature, predation pressure (measured at the site level), and the resulting two‐term interaction as explanatory variables. The phylogenetic tree based on microsatellite markers was incorporated into each model to account for genetic relatedness among populations. When λ = 0, the model is equivalent to a classical linear model, whereas when λ = 1 it accounts for phylogenetic conservatism in trait. We then used an information‐theoretic approach, based on Akaike Information Criteria (AIC) comparisons, to select the model(s) that best fit the data. We considered model(s) that fell within a ΔAIC <4 as “best” model(s) as they would maximize the likelihood of the model while taking into account the number of parameters, and we rejected those with a ΔAIC>4 (Burnham & Anderson, 2002). We ran PGLS models using the *pgls* function from the *caper* R‐package (Orme et al., 2013).

#### 2.7.6 *F*_ST_ */P*_ST_ *comparison*

Finally, we tested whether variability in traits and covariations among populations were higher or not than expected under the hypothesis that differentiation is due to genetic drift only. To do so, we compared *F*_ST_ calculated on neutral genetic markers (corresponding to the level of differentiation among populations expected if genetic drift only is affecting traits) to *P*_ST_ values calculated for each trait and covariation independently. *P*_ST_ is the phenotypic equivalent of the *Q*_ST_ index, although calculated for wild populations when no information on the parental relatedness among individuals is available (Leinonen, Cano, MäKinen, & Merilä, 2006). A *P*_ST_ value higher than the global *F*_ST_ value indicates that phenotypic differentiation among populations is higher than expected by genetic drift only, and that mechanisms such as plasticity and/or selection might explain these differences (Leinonen et al., 2013). The use of F0 individuals allows comparison of natural trait variability and covariations existing among wild populations. However, this approach does not enable to tease apart genetic and plastic contributions to trait variability and covariations. Therefore, *P*_ST_ here represents the level of phenotypic differentiation that is due to both genetic and developmental components. We estimated a *P*_ST_ for each trait as: $\sigma_{\text{B}}^{2}/\sigma_{\text{B}}^{2}+\sigma_{\text{W}}^{2}$ where $\sigma_{\text{B}}^{2}$ and $\sigma_{\text{W}}^{2}$were, respectively, the among‐ and within‐population variance in the considered trait (Leinonen et al., 2013). Among‐ and within‐population variance components were estimated from generalized linear mixed models with the trait as response variable, the intercept as a fixed effect, and the population as a random effect (Leinonen et al., 2013).

In the case of covariations, among‐ and within‐population variance components were calculated in a similar way but with the addition of a random slope, corresponding to the covariable trait (Supporting Information Appendix S2). This allows estimating among‐ and within‐population variance in the covariation between each pair of traits (Mazé‐Guilmo, Blanchet, Rey, Canto, & Loot, 2016). The generalized linear mixed models were run using the *lme4* R‐package (Bates, Maechler, Bolker, & Walker, 2014). We applied a classical bootstrap clustering procedure with 1,000 iterations (Field & Welsh, 2007) to assess the 95% confidence interval for *P*_ST_. We then compared the CI of *P*_ST_ for each trait and each covariation (i.e., 10 *P*_ST_ quantified in total: 4 single traits and 6 covariations among them) to the CI of *F*_ST_. All analyses were performed using R (R Core Team, 2013).

## 3 RESULTS

### 3.1 Trait variability among populations

**Figure 2:**
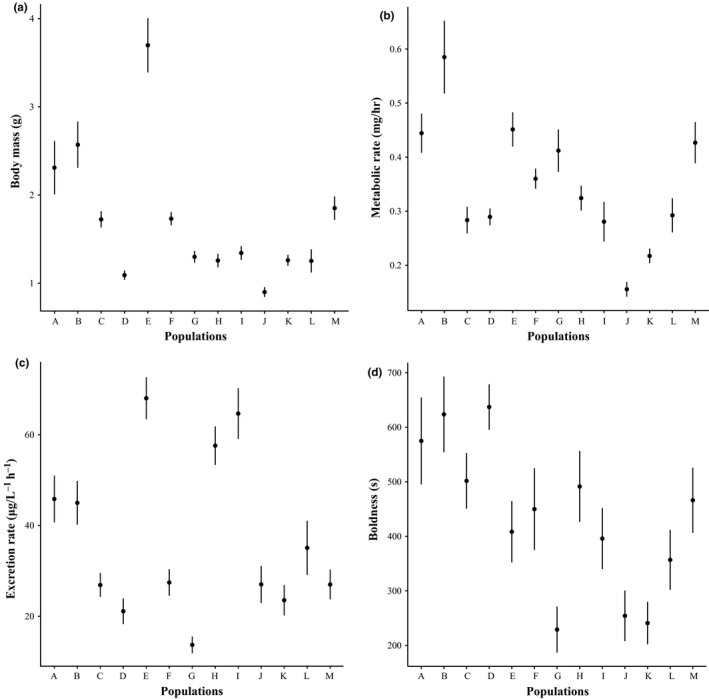
Mean trait values for body mass (a), metabolic rate (b), excretion rate (c), and boldness (d) in function of the population origin of fish. Error bars represent ±1*SE*

**Figure 3:**
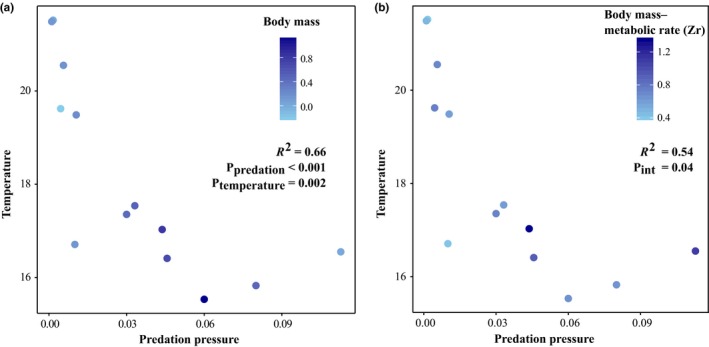
Interaction between temperature (°C) and predation pressure (ind.m^2^) explains the variation in body mass (a), and in the covariation between body mass and metabolic rate (b). The *R* ^2^ and the *p*‐values are extracted from the best models based on AIC selection (see Table 1), and “P_int_” represents the *p*‐value for the interaction between temperature and predation

**Figure 4:**
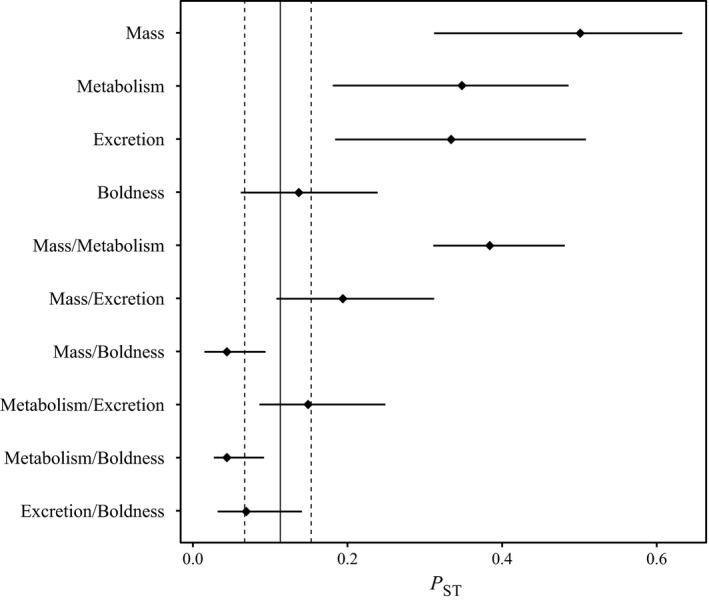
Estimates of *P*_ST_ for each trait (body mass, metabolic rate, excretion rate, and boldness) and for each covariation (body mass–metabolic rate, body mass–excretion rate, body mass–boldness, metabolic rate–excretion rate, metabolic rate–boldness, and excretion rate–boldness), and *F*_ST_ (vertical straight line) on neutral microsatellite markers. Horizontal bars represent 95% confident interval of *P*_ST_ *,* and vertical dotted line represents 95% confident interval of *F*_ST_ that were calculated using cluster bootstrap procedure

Body mass (*F* = 29.859, *df* = 12, 349, *p* < 0.001), metabolic rate (*F* = 14.538, *df* = 12, 350, *p* < 0.001), excretion rate (*F* = 14.842, *df* = 12, 322, *p* < 0.001), and boldness (*F* = 5.179, *df* = 12, 329, *p* < 0.001) were all significantly different among populations (Figure 2). There was no strong evidence for phylogenetic conservatism for any of the traits (see Supporting Information Figure S1): λ was highest for body mass (λ = 0.87) and metabolic rate (λ = 0.74), although none of these values were significantly different from zero (Table 1). Regarding determinants of trait means, the best models explaining body mass included temperature, predation pressure, and their interaction (Table 1). Body mass increases as temperature decreases (negative relationship), and this increase was exacerbated as predation pressure increased (Figure 3a). The model selection for the three other traits led to equivalent models, and the null models were, in all‐three cases, the best models (Table 1). This suggested that metabolic rate, excretion rate, and boldness were neither—or weakly—related to temperature, nor to predation pressure. Finally, the estimates of phenotypic differentiation among populations (*P*_ST_) were high for body mass, metabolic rate, and excretion, and were significantly higher than the level of neutral genetic differentiation (*F*_ST_) (Figure 4). Phenotypic differentiation measured for boldness was not different from what was expected under the drift hypothesis.

**Table 1:** Results of the model selection to explain the variability of functional traits and their covariations among populations. All possible phylogenetic models (PGLS, see the main text) were run for each trait and then compared based on AIC. Bold values represent models that fell in a ΔAIC <4

	λ (*p*‐value)	Models				
		Null	Temperature	Predation	Temperature and predation	Temperature‐by‐predation
Mass	0.87 (0.12)	7.982	7.018	9.997	**0**	**0.194**
Metabolism	0.74 (0.19)	**0**	**1.451**	**1.997**	**2.907**	4.547
Excretion	0 (1)	**0**	**1.016**	**1.67**	**2.952**	4.521
boldness	0.55 (1)	**0**	**1.982**	**1.932**	**3.925**	5.924
Mass–metabolism	0 (1)	4.123	**3.528**	**1.966**	**3.929**	**0**
Mass–excretion	0 (1)	**0**	**1.411**	**1.8**	**0.732**	**1.617**
Mass–boldness	0 (1)	**0**	**1.93**	**1.057**	**2.559**	**2.27**
Metabolism–excretion	0 (1)	**2.719**	4.611	4.64	5.963	**0**
Metabolism–boldness	0 (1)	**0.35**	**1.698**	**0**	**1.757**	**3.332**
Excretion–boldness	0 (1)	**0**	**1.102**	**1.853**	**2.862**	**3.639**

### 3.2 Among population heterogeneity in functional trait syndromes and covariations

**Figure 5:**
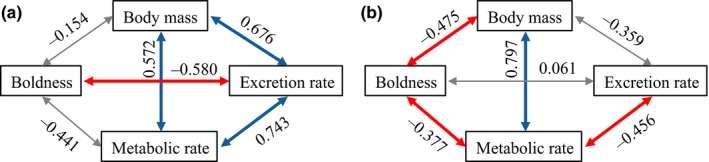
Syndromes of functional traits among populations of European minnow. Populations *F* and *L* were represented as examples in panel (a) and (b), respectively. Blue and red arrows denote significant positive and negative covariances, respectively, while the gray arrow represents nonsignificant covariance. Syndromes in all populations are displayed in Supporting Information Figure S3

We found that populations varied in their syndromes of functional traits since the matrices of covariations were heterogeneous (RMSEA = 0.266, CFI = 0.602, SRMR = 0.263, Supporting Information Figure S2). For instance, the syndrome in the population F was characterized by positive covariations among body mass, metabolic rate, and excretion rate, and a negative covariation between boldness and excretion rate (Figure 5a); whereas population L displayed negative covariations between body mass and boldness, boldness and metabolic rate, and metabolic and excretion rates, while the body mass–metabolic rate covariation was positive (Figure 5b).

**Figure 6:**
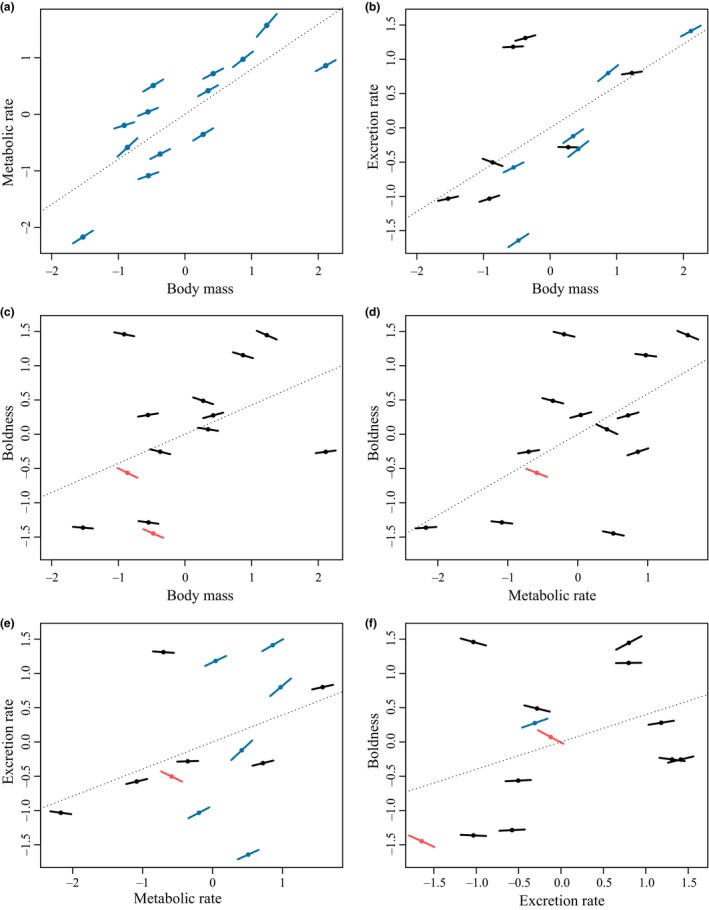
Covariations between each pair of functional traits: (a) body mass–metabolic rate, (b) body mass–excretion rate, (c) body mass–boldness, (d) metabolic rate–excretion rate, (e) metabolic rate–boldness, and (f) excretion rate–boldness. Points represent the average trait value for each population, and lines on points represent the covariations (i.e., the slope) between traits within each population. Blue and red lines indicate significant (α = 0.05) positive and negative covariations, respectively. The dotted lines represent the relationship between traits across the thirteen populations

This was confirmed since we also found strong significant heterogeneity among populations for several trait covariations. In particular, the covariations measured between body mass and excretion rate (*H* = 72.03%, *Q* = 45.837, *df* = 12, *p* < 0.001), between excretion rate and metabolic rate (*H* = 69.20%, *Q* = 41.229, *df* = 12, *p* < 0.001), and between excretion rate and boldness (*H* = 58.26%, *Q* = 31.296, *df* = 12, *p* = 0.002) strongly (and significantly) varied among populations (Figure 6b,e, and f). For instance, the covariation between metabolic and excretion rates was significantly positive for six populations, significantly negative for one population, and nonsignificant for the remaining populations (Figure 6e). The covariations between body mass and metabolic rate, between metabolic rate and boldness, and between body mass and boldness were homogeneous (*p* > 0.052, Figure 6a,c, and d).

We did not find evidence for significant phylogenetic conservatism for any of the covariations (Table 1 and Supporting Information Figure S3). The best models explaining the covariation between body mass and metabolic rate included temperature, predation, and the temperature‐by‐predation interaction term (Table 1). For this covariation, the null model was strongly rejected from the set of best‐supported models (ΔAIC >4), and the results suggested that the strength of the covariation tended to increase as the temperature decreased, and when the predation pressure increased (Figure 3b). Regarding other covariations, models including temperature and predation pressure were not strongly supported by the data as the null models were always selected within the set of models displaying a ΔAIC <4 (Table 1).

Finally, covariation measured between body mass and metabolic rate displayed a *P*_ST_ value that was significantly higher than the global *F*_ST_ value (Figure 4). *P*_ST_ measured for the covariation between body mass and excretion rate was higher than the global *F*_ST_, but the CIs of the two estimates overlapped. For other trait covariations, *P*_ST_ values were not significantly different from the global *F*_ST_ value (Figure 4).

## 4 DISCUSSION

We demonstrated that functional traits, trait covariations, and syndromes they form strongly varied across populations of European minnow sampled in a large riverscape. We further found that multiple processes explained variability in functional traits, their covariations, and hence in syndromes of functional traits. For instance, we found evidence for adaptive mechanisms (plasticity and/or selection) related to water temperature and/or predation for explaining the covariation between body mass and metabolic rate. In parallel, we found that other traits and covariations were consistent with the hypothesis that genetic drift is sufficient to explain variability, which would suggest that even nonadaptive processes could sustain intraspecific variation in functional traits. Finally, we do not detect any evidence of evolutionary conservatism in any of the functional traits nor in their covariations.

We showed that body mass, metabolic rate, and excretion rate differed among populations more than expected by genetic drift only, suggesting trait divergences arose from selection and/or developmental plasticity. Although our design does not allow selection to be teased apart from developmental plasticity, our findings are theoretically sound and may suggest adaptation to environmental conditions since the decrease in body mass with temperature is expected for ectotherms (Daufresne, Lengfellner, & Sommer, 2009). Here, we found that both temperature and predation intensity affected body mass. We can speculate that higher body mass could allow minnows to reach a size refuge from predators, and/or to increase their locomotor performances to escape predators (Domenici, 2001; Villéger et al., 2017). Nonetheless, this result should be interpreted with care since our statistical power is weak and because of collinearity between water temperature and predation. Indeed, we could alternatively argue (based on the visual inspection of biplot, Supporting Information Figure S4) that a quadratic relationship (Supporting Information Figure S4) exists between body mass and predation pressure that we may fail to properly identify because of the small sample size and the collinearity with water temperature (Prunier & Blanchet, 2018). Collinearity can, in some cases, lead to inappropriate conclusions since it is difficult to discriminate the causal links among explicative variables, or because model estimates may be biased (Prunier & Blanchet, 2018; Prunier, Colyn, Legendre, Nimon, & Flamand, 2015). However, since the results are biologically sound, we are confident that body mass is adaptively related to environmental variables. We also found high variability in metabolic and excretion rates, which were also likely driven by adaptive mechanisms (Figure 4). Nonetheless, we failed to detect the environmental pressures driving divergences in these two traits. The variability in excretion rate probably stands in trophic and stoichiometric factors, such as trophic niche, elemental composition of resources, or allochthonous nutrient inputs (El‐Sabaawi, Warbanski, Rudman, Hovel, & Matthews, 2016; Evangelista et al., 2017), which could be characteristic of each geographical site. Hence, measuring stoichiometric variability of individuals and populations would benefit to infer hypotheses regarding variability in excretion rate.

We found that traits can not only vary among populations, but also that functional traits formed different syndromes among populations of European minnow. Indeed, the sets of covariations were different among populations, and multiple patterns were identified, with some trait covariations being more robust than others. For instance, the allometric relationships between body mass and metabolic rate, and between body mass and excretion rate were both positive across all populations, but the former was homogeneous among populations (i.e., stable), whereas the latter was heterogeneous and hence more flexible among populations (Figure 6). Similarly, the covariation between excretion rate and boldness was flexible, confirming that relationships between behavioral and physiological traits can be complex (Killen et al., 2013). These various functional trait covariations among populations subsequently generated variability in syndromes. Such variability has been documented in behavioral traits (Dingemanse et al., 2007) and morphological traits (Berner, Stutz, & Bolnick, 2010), but rarely among multiple types of traits. The various biological mechanisms—such as pleiotropy or allometry—underlying the links among traits might therefore be modulated differently among populations, resulting in difference of syndromes (Peiman & Robinson, 2017). Hence, it would be worth further investigating the biological mechanisms driving trait covariations to better appraise the variability of functional syndromes (Killen, Atkinson, & Glazier, 2010; Raffard et al., 2017).

Although we detected variability in syndromes of functional traits, the lack of determinants (i.e., temperature or predation) and the low *P*_ST_ values for most covariations suggest that a non‐negligible part of the heterogeneity in syndromes variability may—in our case—arise from the effect of genetic drift. Actually, the relationship between body mass and metabolic rate was the only covariation whose variability was likely driven by adaptive mechanisms. Indeed, as revealed by the *P*_ST_/*F*_ST_ analysis and the trait–environment analysis, we found evidence that selection and/or plasticity associated with predation pressure and water temperature may drive variation observed among populations. Previous works have reported variability in the allometric relationship between body mass and metabolic rate at both inter‐ and intraspecific levels in many organisms (Bokma, 2004; Glazier, 2005; Seibel, 2007). Here, covariations increase as temperature decreases and predation increases (Figure 2b). Although this should be interpreted with care (see statistical caution above), the metabolic allometry might vary allowing individuals to optimize energetic efficiency under different environmental constraints (Glazier, 2005; Killen et al., 2010). Fish can notably adapt their lifestyle to increase or decrease their energetic assimilation in order to cope with biotic and abiotic constraints, such as predation (Killen et al., 2010). This confirms that trait architecture within populations can be complex, and—in some cases—allow individuals to adapt/acclimatize to their environment (Peiman & Robinson, 2017).

To conclude, we found that syndromes in functional traits can strongly vary among populations, and that both adaptive (natural selection and/or plasticity) and nonadaptive processes (genetic drift) are driving intraspecific heterogeneity in these syndromes. Since functional traits can affect ecological processes (Lavorel & Garnier, 2002; Raffard et al., 2017; Violle et al., 2007), the variability in functional syndromes may exert puzzling effects on ecological processes. For instance, the variability in covariations involving excretion rate may have implications for the dynamics of nutrient recycling and ecological stoichiometry (Atkinson, Capps, Rugenski, & Vanni, 2017; Vanni, 2002); while in some populations, large individuals should excrete a high quantity of nitrogen, they should excrete a low quantity of nitrogen in other populations, with potential consequences for primary production (Evangelista et al., 2017; McIntyre et al., 2008). Variability of syndromes may have further ecological effects through trophic mechanisms since individuals with different functional traits may have different trophic niches (Villéger et al., 2017). Trophic variability can subsequently affect community structure and ecosystem functioning (Des Roches, Shurin, Schluter, & Harmon, 2013). Further studies should aim to experimentally test how heterogeneity in functional syndromes is acting on ecological dynamics.

## CONFLICT OF INTEREST

None declared.

## AUTHOR CONTRIBUTIONS

AR, SB, FS, and JC designed the study. AR and SB performed fieldwork, and AR performed trait measurement. GL carried out the processing of genetic samples. JGP analyzed genetic data and AR performed statistical analyses. All authors interpreted and discussed the results. AR and SB wrote the first draft of the paper. All authors corrected and improved the paper, and approved this version of the manuscript.

## Data Availability

Data are available on Figshare. https://doi.org/10.6084/m9.figshare.7623854.
