# Supplementary material for: Variability of functional traits and their syndromes in a freshwater fish species (Phoxinus phoxinus): The role of adaptive and nonadaptive processes
Source: Ecol Evol. 2019 Feb 14;9(5):2833–46. doi: 10.1002/ece3.4961 (PMC6405509; doi:10.1002/ece3.4961)
Supplement: Supplementary file 1 [file ECE3-9-2833-s001.docx]

Variability of functional traits and their syndromes in a freshwater fish species (*Phoxinus phoxinus*): the role of adaptive and non-adaptive processes

Allan Raffard, Julien Cucherousset, Jérôme G. Prunier, Géraldine Loot, Frédéric Santoul and Simon Blanchet

**Appendix S1**

Eighteen microsatellites were amplified in two multiplex PCR as described below.

Locus CtoG-075 was discarded from statistical analyses as we identified evidence for null alleles (see main text for details)

| **MINNOWS MULTIPLEX 1** | |  |  |  |
| --- | --- | --- | --- | --- |
| **Locus** | **Accession number** | **Reference** | **Allele size range** | **Forward primer (5 ′– 3 ′)** |
| CypG9 | AY439127 | 1 | 107-115 | GCAGTCACGTATTAAGGCGAGCAG |
| Rru4 | AB112740 | 2 | 163-205 | TAAGCAGTGACCAGAATCCA |
| LleA-071 | FJ601719 | 3 | 340-371 | GTCTTAGATTGTGTAGCGGG |
| Lsou8 | EF209003 | 4 | 175-200 | GCGGTGAACAGGCTTAACTC |
| BL1-153 | FJ468350 | 5 | 217-284 | GCACAGCTCTAATCGGTCACT |
| Ppro132 | AY254354 | 6 | 113-123 | GCATTTCCTTTTGCTTGTAAGTCTCAA |
| LleB-072 | FJ601720 | 3 | 150-174 | TCATTAGGGAGGCTGCTTATTC |
| Ca3 | AF277575 | 7 | 215-311 | GGACAGTGAGGGACGCAGAC |
| CtoA-247 | GU254031 | 8 | 162-182 | TGCAAACATATAAACTGAAACAAGG |
| CtoG-075 | GU254035 | 8 | 217-225 | TCATTTGGATAACAATCCATCATCAC |
|  |  |  |  |  |
|  |  |  |  |  |
| **Locus** | **Reverse primer (5 ′– 3 ′)** | **Fuorescent dye** | **Observations** |  |
| CypG9 | GAGCGGACTCTCAGGCACCTACC | FAM | / |  |
| Rru4 | CAAAGCCTCAAAAGCACAA | FAM | / |  |
| LleA-071 | ACTTCAGTTACTAAGAGATTAGTGA | FAM | / |  |
| Lsou8 | TAGGAACGAAGAGCCTGTGG | HEX | / |  |
| BL1-153 | TATGGTCAAACACGGGTCAA | HEX | / |  |
| Ppro132 | GGTTTAACCCGATCAATGGCTGTGC | A550 | / |  |
| LleB-072 | CCTTTTCAACAATTTGTCACGG | A550 | / |  |
| Ca3 | TCTAGCCCCCAAATTTTACGG | A550 | / |  |
| CtoA-247 | GCAGGTATATTCCCAGCC | A565 | / |  |
| CtoG-075 | ACTATGTTAGCATCCACACC | A565 | Null alleles |  |

| **Locus** | **Reverse primer (5 ′– 3 ′)** | **Fuorescent dye** | **Observations** |
| --- | --- | --- | --- |
| CypG9 | GAGCGGACTCTCAGGCACCTACC | FAM | / |
| Rru4 | CAAAGCCTCAAAAGCACAA | FAM | / |
| LleA-071 | ACTTCAGTTACTAAGAGATTAGTGA | FAM | / |
| Lsou8 | TAGGAACGAAGAGCCTGTGG | HEX | / |
| BL1-153 | TATGGTCAAACACGGGTCAA | HEX | / |
| Ppro132 | GGTTTAACCCGATCAATGGCTGTGC | A550 | / |
| LleB-072 | CCTTTTCAACAATTTGTCACGG | A550 | / |
| Ca3 | TCTAGCCCCCAAATTTTACGG | A550 | / |
| CtoA-247 | GCAGGTATATTCCCAGCC | A565 | / |
| CtoG-075 | ACTATGTTAGCATCCACACC | A565 | Null alleles |
|  |  |  |  |
|  |  |  |  |
|  |  |  |  |
|  |  |  |  |
|  |  |  |  |
| **Locus** | **Primer mix (total 100µM each)** | |  |
|  | **Forward (µl)** | **Reverse (µl)** |  |
| CypG9 | 10 | 10 |  |
| Rru4 | 16 | 16 |  |
| LleA-071 | 20 | 20 |  |
| Lsou8 | 6 | 6 |  |
| BL1-153 | 6 | 6 |  |
| Ppro132 | 20 | 20 |  |
| LleB-072 | 6 | 6 |  |
| Ca3 | 20 | 20 |  |
| CtoA-247 | 6 | 6 |  |
| CtoG-075 | 10 | 10 |  |
|  |  |  |  |
|  | **PCR MIX** | **Volume (µL) X1** |  |
|  | H2O | 3.94 |  |
|  | Primer mix | 0.06 |  |
|  | Qiagen multiplex PCR Master Mix | 5 |  |
|  | DNA | 1 |  |
|  |  |  |  |
|  |  |  |  |
| **Cycling conditions** |  |  |  |
| 95°C | \| 15 min \| \| --- \| |  |  |
| 94°C | 30 s |  |  |
| 56°C | 90 s | 35 cycles |  |
| 72°C | 60 s |  |  |
| 60°C | 45 min |  |  |
| 10°C | ∞ |  |  |

| **MINNOWS MULTIPLEX 2** | |  |  |  |
| --- | --- | --- | --- | --- |
|  |  |  |  |  |
| **Locus** | **Accession number** | **Reference** | **Allele size range** |  |
| BL1-44 | FJ468355 | 5 | 110-160 |  |
| BL1-84 | FJ468346 | 5 | 177-205 |  |
| LleC-090 | FJ601722 | 3 | 215-350 |  |
| LC27 | EF362792 | 9 | 104-200 |  |
| LceC1 | AY962241 | 10 | 93-140 |  |
| MFW1 | AY703054 | 11 | 163-290 |  |
| Rhca20 | DQ106915 | 5 | 110-130 |  |
| Lsou5 | EF209002 | 4 | 187-260 |  |
| BL1-98 | FJ468349 | 5 | 270-340 |  |
|  |  |  |  |  |
| **Locus** | **Forward primer (5 ′– 3 ′)** | **Reverse primer (5 ′– 3 ′)** | **Fuorescent dye** | **Observations** |
| BL1-44 | AAGACCAGCATGTGCTT | ACATAGACTAACCAGTTTCACTT | FAM | / |
| BL1-84 | CATTACTACGGCAACCACAT | GCGAAAAGGAAAGAGACTGA | FAM | / |
| LleC-090 | TCAGACACAACTAACCGACC | GGCGCTGTCCAGAACTGA | FAM | / |
| LC27 | TCCAGTTCTTCCTTCCTAATT | GCGGAGGGAGAGTATGTCAA | HEX | / |
| LceC1 | AGGTGTTGGTTCCTCCCG | TGTTATCTCGGTTTCACGAGC | A565 | / |
| MFW1 | GTCCAGACTGTCATCAGGAG | GAGGTGTACACTGAGTCACGC | A565 | / |
| Rhca20 | CTACATCTGCAAGAAAGGC | CAGTGAGGTATAAAGCAAGG | A550 | / |
| Lsou5 | CTGAAGAAGACCCTGGTTCG | CCCACATCTGCTGACTCTGAC | A550 | / |
| BL1-98 | ATTGTTTTCATTTTGTCAG | CCGAGTGTCAGAGTTATT | A550 | / |

| **Locus** | **Primer mix (total 100µM each)** | |
| --- | --- | --- |
|  | **Forward (µl)** | **Reverse (µl)** |
| BL1-44 | 15 | 15 |
| BL1-84 | 20 | 20 |
| LleC-090 | 16 | 16 |
| LC27 | 10 | 10 |
| LceC1 | 40 | 40 |
| MFW1 | 60 | 60 |
| Rhca20 | 6 | 6 |
| Lsou5 | 40 | 40 |
| BL1-98 | 60 | 30 |
|  |  |  |
| **PCR MIX** | | **Volume (µL) X1** |
| H2O | | 3.867 |
| Primer mix | | 0.133 |
| Qiagen multiplex PCR Master Mix | | 5 |
| DNA | | 1 |

|  | References |
| --- | --- |
|  |  |
| 1 | Baerwald, M. R., & May, B. (2004). Characterization of microsatellite loci for five members of the minnow family Cyprinidae found in the Sacramento-San Joaquin Delta and its tributaries. Molecular Ecology Notes, 4(3), 385–390. doi:10.1111/j.1471-8286.2004.00661.x |
| 2 | Barinova, A., Yadrenkina, E., Nakajima, M., & Taniguchi, N. (2004). Identification and characterization of microsatellite DNA markers developed in ide Leuciscus idus and Siberian roach Rutilus rutilus. Molecular Ecology Notes, 4(1), 86–88. doi:10.1046/j.1471-8286.2003.00577.x |
| 3 | Dubut, V., Martin, J.-F., Gilles, A., Van Houdt, J. K. J., Chappaz, R., & Costedoat, C. (2009). Isolation and characterization of polymorphic microsatellite loci for the dace complex: *Leuciscus leuciscus* (Teleostei: Cyprinidae). Molecular Ecology Resources, 9(4):1179-83. doi: 10.1111/j.1755-0998.2009.02594.x |
| 4 | Muenzel, F. M., Sanetra, M., Salzburger, W., & Meyer, A. (2007). Microsatellites from the vairone Leuciscus souffia (Pisces: Cyprinidae) and their application to closely related species. Molecular Ecology Notes, 7(6), 1048–1050. doi:10.1111/j.1471-8286.2007.01772.x |
| 5 | Dubut, V., Martin, J.-F., Costedoat, C., Chappaz, R., & Gilles, A. (2009). Isolation and characterization of polymorphic microsatellite loci in the freshwater fishes Telestes souffia and Telestes muticellus (Teleostei: Cyprinidae). Molecular Ecology Resources, 9(3), 999–1001. doi:10.1111/j.1755-0998.2009.02536.x |
| 6 | Bessert, M. L., & Orti, G. (2003). Microsatellite loci for paternity analysis in the fathead minnow, Pimephales promelas (Teleostei: Cyprinidae). Molecular Ecology Notes, 3(4), 532–534. doi:10.1046/j.1471-8286.2003.00501.x |
| 7 | Dimsoski, P., Toth, G. P., & Bagley, M. J. (2000). Microsatellite characterization in central stoneroller Campostoma anomalum (Pisces : Cyprinidae). Molecular Ecology, 9(12), 2187–2189. |
| 8 | Dubut, V., Sinama, M., Martin, J.-F., Meglécz, E., Fernandez, J., Chappaz, R., … Costedoat, C. (2010). Cross-species amplification of 41 microsatellites in European cyprinids: A tool for evolutionary, population genetics and hybridization studies. BMC Research Notes, 3(1), 135. doi:10.1186/1756-0500-3-135 |
| 9 | VyskočIlová, M., šImková, A., & Martin, J.-F. (2007). Isolation and characterization of microsatellites in Leuciscus cephalus (Cypriniformes, Cyprinidae) and cross-species amplification within the family Cyprinidae. Molecular Ecology Notes, 7(6), 1150–1154. doi:10.1111/j.1471-8286.2007.01813.x |
| 10 | Larno, V., Launey, S., Devaux, A., & Laroche, J. (2005). Isolation and characterization of microsatellite loci from chub Leuciscus cephalus (Pisces: Cyprinidae). Molecular Ecology Notes, 5(4), 752–754. doi:10.1111/j.1471-8286.2005.01052.x |
| 11 | Crooijmans, R. P. M. A., Poel, J. J. V. der, Groenen, M. A. M., Bierbooms, V. A. F., & Komen, J. (1997). Microsatellite markers in common carp (Cyprinus carpio L.). Animal Genetics, 28(2), 129–134. doi:10.1111/j.1365-2052.1997.00097.x |
