# Supplementary material for: Variability of functional traits and their syndromes in a freshwater fish species (Phoxinus phoxinus): The role of adaptive and nonadaptive processes
Source: Ecol Evol. 2019 Feb 14;9(5):2833–46. doi: 10.1002/ece3.4961 (PMC6405509; doi:10.1002/ece3.4961)
Supplement: Supplementary file 2 [file ECE3-9-2833-s002.docx]

**Supplementary information**

**Variability of functional traits and their syndromes in a freshwater fish species (*Phoxinus phoxinus*): the role of adaptive and non-adaptive processes**

A. Raffard, J. Cucherousset, J. G. Prunier, G. Loot, F. Santoul, and S. Blanchet

**Figure S1.** Mean trait values (scaled to the mean) in function of the position of the populations in the phylogenetic tree constructed based on genetic distance using neutral microsatellite markers.

**Figure S2.** Syndromes of functional traits in each of the 13 populations (panels A to G correspond to the populations with the same code). Blue and red arrows denote respectively significant positive and negative covariance, while the grey arrow represents non-significant covariance.

**Figure S3.** Covariation values (transformed into a *Zr* and scaled to the mean) in function of the position of the populations in the phylogenetic tree constructed based on genetic distance using neutral microsatellite markers

**Figure S4.** Mean body mass (log-transformed) for each population in function of temperature **(a)** and predation pressure **(b)**.

**Appendix S2.** Construction of the linear mixed effects models (LMM) used to calculated *Pst* of covariations*. Pst* were calculated as: σ^2^_Bs_ / (σ^2^_tot_) where σ^2^_Bs_ is the among variation in the slope and σ^2^_tot_ is the total amount of variance (Mazé-Guilmo *et al.* 2016). Traits were scaled to the mean. LMM were run using the lme4 package in R (Bates *et al.* 2014).

| *Y* | *X* | Random effect | Random slope |
| --- | --- | --- | --- |
| Metabolic rate | Intercept | Population | Body mass |
| Excretion rate | Intercept | Population | Body mass |
| Excretion rate | Intercept | Population | Metabolic rate |
| Excretion rate | Intercept | Population | Boldness |
| Boldness | Intercept | Population | Body mass |
| Boldness | Intercept | Population | Metabolic rate |

**References**

Bates, D., Maechler, M, Bolker, B & Walker, S. (2014). lme4: Linear mixed-effects models using Eigen and S4. R package version 1.1-7.

Mazé-Guilmo, E., Blanchet, S., Rey, O., Canto, N. & Loot, G. (2016). Local adaptation drives thermal tolerance among parasite populations: a common garden experiment. *Proc. R. Soc. B Biol. Sci.*, 283, 20160587.
